# Return to Work after Upper or Lower Extremity Long Bone Fractures: Social Factors in a Japanese Multicenter Cohort Study

**DOI:** 10.31662/jmaj.2024-0157

**Published:** 2024-11-25

**Authors:** Keisuke Ishii, Hiroyuki Oka, Koichi Inokuchi, Takashi Maehara, Akiro Higashikawa, Yoshiaki Sasashige, Hiroaki Konishi, Fumitake Nakajima, Yoshimasa Tomita, Shingo Nobuta, Yoji Mikami

**Affiliations:** 1 Trauma and Reconstruction Center, Teikyo University Hospital, Tokyo, Japan; 2 22^nd^ Century Medical and Research Center, The University of Tokyo Hospital, Tokyo, Japan; 3 Trauma Center, Saitama Medical Center, Saitama Medical University, Saitama, Japan; 4 Department of Orthopaedic Surgery, Kagawa Rosai Hospital, Kagawa, Japan; 5 Department of Orthopaedic Surgery, Kanto Rosai Hospital, Kanagawa, Japan; 6 Department of Orthopaedic Surgery, Chugoku Rosai Hospital, Hiroshima, Japan; 7 Department of Orthopaedic Surgery, Nagasaki Rosai Hospital, Nagasaki, Japan; 8 Department of Orthopaedic Surgery, Chiba Rosai Hospital, Chiba, Japan; 9 Department of Orthopaedic Surgery, Tokyo Rosai Hospital, Tokyo, Japan; 10 Department of Orthopaedic Surgery, Tohoku Rosai Hospital, Miyagi, Japan; 11 Department of Orthopaedic Surgery, Yokohama Rosai Hospital, Kanagawa, Japan

**Keywords:** Return to work, Fracture, Extremity, Chronic pain

## Abstract

**Introduction:**

Fractures cause serious impediments to employment. In Japan, there is insufficient evidence regarding social factors, such as nonregular employment, and return to work (RTW) after an injury. This study aimed to determine the association between social factors and RTW following injury.

**Methods:**

This multicenter cohort study was conducted from 2015 to 2018 and included 674 patients aged 18-65 years who were workers at the time of injury and underwent surgery for long bone fractures of the upper or lower extremities. The primary outcome was the RTW rate within 2 years following injury. Data on RTW at 6 months, 1 year, and 2 years were collected. Observational data following RTW were not included. The association between RTW at 6 months and within 2 years following injury and social factors were evaluated via logistic regression and Cox proportional hazards regression analyses, respectively, after adjusting for patient- and fracture-related factors.

**Results:**

Overall, 525 (77.9%) and 602 patients (89.3%) resumed work at 6 months and within 2 years, respectively, following injury. Physical labor, open fractures, and chronic pain were associated with the RTW at both 6 months and within 2 years. However, nonregular employment and workers’ compensation insurance were only associated with RTW at 6 months. Social factors were associated with the RTW rate at 6 months but not within 2 years following injury.

**Conclusions:**

Approximately 10% of patients with fractures did not resume work within 2 years following injury. This analysis points to social factors as a risk for delaying early RTW and has implications for interventions at the policy level.

## Introduction

Musculoskeletal injuries, such as extremity fractures, have profound impact on the lives of patients. Among workers, fractures often result in prolonged absence from work, which causes social and economic losses. In Japan, injury is one of the primary reasons for long-term absence, along with mental illness and cancer ^(1)^. Thus, return to work (RTW) following a fracture is important for both the individual and society.

Several factors, including individual (job and injury characteristics), therapeutic (surgical treatment and rehabilitation), social (employment status and insurance characteristics), and social macroeconomic (compensation programs, litigation, or current unemployment rates) factors, are predictors of work resumption ^(2)^. However, systematic reviews on RTW following orthopedic trauma are inconclusive owing to the limited number of measured predictors ^(3)^. The National Databank, which is a trauma database in Japan, showed in 2019 that musculoskeletal trauma accounts for 47% of all cases of injury ^(4)^. As this type of injury is prevalent, knowledge on RTW following trauma is important. However, no large-scale studies on RTW following a musculoskeletal injury have been conducted in Japan. Furthermore, epidemiological findings are lacking, and factors that inhibit workers from RTW remain unclear. If these factors can be identified, interventions may increase the chances or provide early support for treatment that may accelerate workers’ RTW.

Fractures commonly occur in the extremities, pelvis, and spine. Traumatic injuries to the hands and feet significantly vary from minor to severe, and the injury may be complex. Pelvic and spinal injuries are usually serious and are accompanied by injuries to other organs. Spinal injuries may be treated with other services, such as neurosurgery or spine surgery, as these are also associated with neurological damage, which can make it difficult to regain the ability to perform activities of daily living. Contrarily, fractures of the extremities are generally treated in the orthopedic surgery department, and data analysis is more straightforward for these fractures compared with that for fractures of the hand, foot, pelvis, or spine. Thus, using a database shared among nine centers in Japan, RTW following long bone fractures of the extremities was evaluated to identify the factors associated with RTW at 2 years following a fracture.

## Materials and Methods

**Figure 1.**
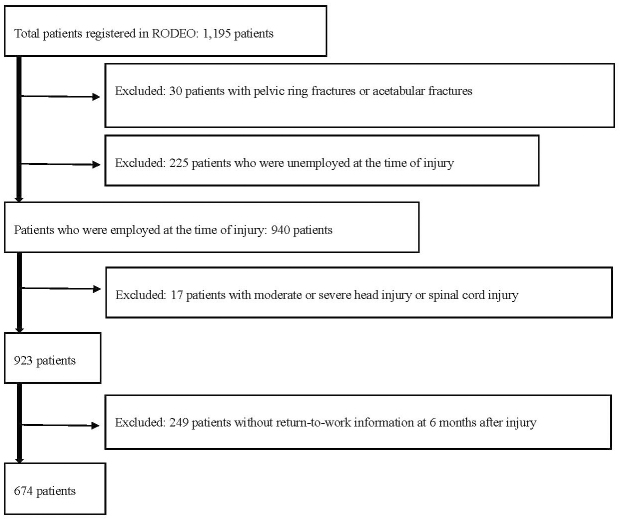
The process of selecting the study population. Patient demographics BMI: body mass index, FRI: fracture-related infection within 90 days following injury, NRS: numeric rating scale. *Classification of open fractures is indicated by the number of fractures, and there are duplicate cases. ** Patients with fractures of the femur, tibia, or fibula were classified as “lower extremity” even if accompanied with an upper extremity fracture

A prospective cohort observational study was conducted at eight national centers and one university hospital, which are widely distributed among urban and rural areas in Japan. A review of the Rosai Orthopedic Trauma Database for Exploratory Outcomes, which was approved by the respective hospital ethics committees, identified 1,195 patients aged 18-65 years who underwent surgery for long bone fractures of the extremities (AO/OTA classification 11, 12, 13, 21, 22, 23, 31, 32, 33, 41, 42, 43, 44) between November 2015 and July 2018. Meanwhile, 30 patients with concomitant pelvic fractures, including pelvic ring fractures or acetabular fractures (AO/OTA classification 61, 62), treated with surgery were excluded. Among the identified patients, 940 were employed at the time of injury, of whom 17 were excluded as they also had moderate-to-severe head injury (Glasgow Coma Scale <13 or underwent open cranial surgery) or spinal cord injury (American Spinal Injury Association Impairment Scale A, B, C, or D), which was likely to have had a greater impact on RTW than fractures. Of the remaining 923 patients, 674 who were followed up for at least 6 months to 2 years following injury and from whom information on RTW was received 6 months following injury were included in the study (Figure 1). The primary outcome was RTW at 2 years following injury. Even if it was not the same job as before, if a patient with fracture returned to work, it was defined as RTW. Data regarding RTW were collected at 6 months, 1 year, and 2 years following injury. Observational data after RTW were not included in the study. The followup rates at 6 months and 2 years were 73.0% and 69.3%, respectively.

The following factors were considered owing to their possible association with RTW: sex, age, obesity (body mass index ≥ 25 kg/m^2^) ^(5)^, and currently smoking; occupational characteristics of physical labor ^(6)^; social factors of regular employees and workers’ compensation insurance (as these persons have more generous guarantees for leave); fracture characteristics, such as open fracture, lower extremity fracture ^(7)^, and fracture-related infection (FRI; defined as occurring within 90 days after injury following surgery) ^(8)^; and chronic pain at 6 months following injury, which was evaluated using a numeric rating scale (NRS). Workers’ compensation insurance was applied to patients who were injured because of labor accidents. The NRS score for chronic pain treated as a binary variable was graded as follows: ≥4 indicated moderate or severe pain according to the National Comprehensive Cancer Network guidelines ^(9)^, and ≤3 indicated mild or no pain.

To examine factors associated with RTW at 6 months following injury, univariate analysis was conducted with these factors as explanatory variables and RTW as the objective variable. Multivariate logistic regression analysis of the association between significant factors in the univariate analysis and RTW at 6 months following injury was then conducted. Moreover, to examine factors associated with RTW at 2 years following injury, log-rank tests were conducted with these factors as explanatory variables and RTW as the objective variable. Factors with *P*-value <0.05 were analyzed using Cox proportional hazards regression. All statistical analyses were conducted using IBM SPSS Statistics ver. 24.0 (IBM Corp., Armonk, NY, USA). The study was approved by the ethics committees of the nine participating centers and was funded by the Ministry of Health, Labor and Welfare. It was also approved by the Institutional Review Board of all participating institutions (Code Number 5: Japan Organization of Occupational Health and Safety, Code Number 19-289: Teikyo University). Due to the prospective observational design, the committee gave the patients a chance to opt out and waived the requirement for individual consent.

## Results

Overall, 674 patients (439 men, 235 women) with an average age of 47.0 years were included in the study. Of them, 76 had multiple fractures, resulting in 750 total fractures. The background characteristics of the patients are presented in Table 1. In patients with multiple fractures, fracture characteristics were classified as “yes” if the patient had at least one fracture that was an open fracture, lower extremity fracture, or FRI. At 6 months, 1 year, and 2 years following injury, 525 (77.9%), 574 (85.2%), and 602 (89.3%), respectively, returned to work.

**Table 1.** Patient Demographics.

Parameter	Cases (n = 674) (%)
Age, mean (SD)	47.0 (11.9)
Sex (M/F)	439/235 (65.0%/35%)
Obesity (BMI ≥ 25 kg/m^2^)	Yes: 191 (28.3%)
	No: 483 (71.7%)
Current smoking	Yes: 220 (32.6%)
	No: 454 (67.4%)
Occupation prior to injury	
Physical labor	220 (32.8%)
Others	454 (67.2%)
Nonregular employment
	Yes 192 (28.5%)
	No 482 (71.5%)
Insurance
Worker’s accident compensation	222 (33.0%)
Others	452 (67.1%)
	Medical health insurance: 406
	Automobile liability insurance: 41
	Public assistance: 3
	Unknown: 2
Open fracture	Yes: 105 (15.6%)
	Gustillo*
	Ⅰ: 13 fractures
	Ⅱ: 48 fractures
	ⅢA: 27 fractures
	ⅢB: 15 fractures
	ⅢC: 12 fractures
	Total: 115 fractures
	No: 569 (84.4%)
Lower extremity	Yes: 407 (60.4%)**
	No: 267 (39.6%)
	Humerus: 78 fractures
	Radius or ulna: 222 fractures
	Femur: 124 fractures
	Tibia or fibula: 326 fractures
	Total: 750 fractures
FRI	Yes: 18 (2.7%)
	No: 656 (97.3)
Chronic pain at 6 months after injury (NRS ≥ 4)	Yes: 143 (21.2)
	No: 529 (78.5)
	Unknown: 2 (0.3)

BMI: body mass index, FRI: fracture-related infection within 90 days after injury, NRS: numeric rating scale. *Classification of open fractures is indicated by the number of fractures, and there are duplicate cases. ** Patients with fractures of the femur, tibia, or fibula were classified as “lower extremity” even if accompanied with an upper extremity fracture.

At 6 months following injury, univariate analysis revealed that current smoking, physical labor, nonregular employment, workers’ compensation insurance, open fractures, lower extremity fracture, FRI, and chronic pain were significantly negatively associated with RTW (Table 2). Furthermore, physical labor, nonregular employment, workers’ compensation insurance, open fractures, FRI, and chronic pain remained significant following multivariate logistic regression analysis (Table 3).

**Table 2.** Characteristics and Univariate Analyses for RTW at 6 Months Postinjury.

Characteristics (n = 674)	Odds ratio	95% CI	*p*-value
Age			.077
Sex (men)	0.79	0.52-1.19	.284
Obesity (BMI ≥25 kg/m^2^)	0.93	0.61-1.42	.758
Current smoking	0.58	0.39-0.86	.006
Diabetes mellitus	0.92	0.39-2.40	.833
Physical labor	0.29	0.19-0.43	<.001
Non-regular employment	0.58	0.39-0.87	.007
Worker’s accident compensation insurance	0.48	0.32-0.71	<.001
Open fracture	0.28	0.18-0.45	<.001
Lower extremity fracture	0.66	0.44-0.99	.0037
FRI	0.17	0.06-0.49	<.001
Chronic pain at 6 months post-injury (NRS ≥4)	0.39	0.25-0.60	<.001

RTW: return to work, BMI: body mass index, CI: confidence interval, FRI: fracture-related infection within 90 days after injury, NRS: numeric rating scale

**Table 3.** Characteristics and Multivariate Logistic Analyses with RTW at 6 Months Postinjury.

Characteristics (n = 674)	Odds ratio	95% CI	*p*-value
Current smoking	0.71	0.47-1.07	.100
Physical labor	0.36	0.23-0.54	<.001
Non-regular employment	0.47	0.31-0.72	<.001
Worker’s accident compensation insurance	0.61	0.40-0.93	.023
Open fracture	0.36	0.22-0.58	<.001
Lower extremity fracture	0.66	0.43-1.01	.056
FRI	0.31	0.11-0.93	.036
Chronic pain at 6 months post-injury (NRS ≥4)	0.41	0.27-0.64	<.001

RTW: return to work, CI: confidence interval, FRI: fracture-related infection within 90 days after injury, NRS: numeric rating scale

The log-rank trend test showed that physical labor, open fractures, FRI, and chronic pain at 6 months postinjury were significantly associated with RTW within 2 years postinjury (Table 4). Cox proportional hazards regression analysis revealed that physical labor, open fractures, and chronic pain at 6 months postinjury were significantly negatively associated with RTW within 2 years postinjury (Table 5).

**Table 4.** Characteristics and Log-Rank Trend Tests for RTW within 2 Years Post Injury.

Characteristics (n = 674)	Observed cases (%)	*p*-value
Age		.28
Sex (men)	387 (88.2)	.460
Obesity (BMI ≥ 25 kg/m^2^)	172 (90.1)	.910
Current smoking	195 (88.6)	.440
Diabetes mellitus	27 (79.4)	.450
Physical labor	183 (83.2)	.010
Nonregular employment	161 (83.9)	.130
Worker’s accident compensation insurance	194 (87.4)	.090
Open fracture	81 (77.1)	.004
Lower extremity	358 (88.0)	.320
FRI	9 (50.0)	.010
Pain at 6 months postinjury (NRS ≥ 4)	114 (79.7)	.015

RTW: return to work, BMI: body mass index, CI: confidence interval, FRI: fracture-related infection within 90 days after injury, NRS: numeric rating scale

**Table 5.** Characteristics and Cox Proportional Hazards Regression Analysis for RTW within 2 Years Postinjury.

Characteristics (n = 674)	Hazard ratio	95% CI	*p*-value
Age	0.996	0.99-1.00	.263
Sex (men)	0.99	0.81-1.21	.928
Current smoking	0.96	0.80-1.15	.636
Diabetes mellitus	0.86	0.58-1.27	.449
Physical labor	0.83	0.68-1.00	.0049
Non regular employment	0.87	0.72-1.06	.178
Worker’s accident compensation insurance	0.93	0.78-1.12	.452
Open fracture	0.76	0.59-0.97	.029
Lower extremity	0.93	0.79-1.10	.416
FRI	0.57	0.290-1.116	.101
Pain at 6 months post-injury (NRS≥4)	0.81	0.656-0.993	.043

RTW: return to work, CI: confidence interval, FRI: fracture-related infection within 90 days after injury, NRS: numeric rating scale

## Discussion

RTW within 2 years postinjury was investigated in 674 patients who underwent surgery for long bone fractures of the extremities. The RTW rate at 6 months postinjury was 77.9% and was associated with physical labor, nonregular employment, workers’ compensation insurance, open fractures, FRI, and chronic pain at 6 months postinjury. Meanwhile, the RTW rate within 2 years postinjury was 89.3% and was associated with physical labor, open fractures, and chronic pain at 6 months postinjury. Previous studies reported RTW rates of 79% following humeral diaphyseal fracture surgery ^(10)^ and 72% 1 year following femoral and tibial shaft fractures ^(11)^. Although this study included fractures in both the upper and lower extremities, the results were similar to those previously reported despite the different locations of the studies.

No association was observed between smoking and RTW after a fracture, consistent with the report of Busse et al. ^(12)^. Although smoking is reportedly negatively associated with bone union ^(13)^, an analysis of RTW predictors in 334 cases following motor vehicle-related orthopedic trauma did not reveal an association between smoking and RTW ^(14)^. Similarly, multivariate logistic analyses and Cox proportional hazards regression analysis did not show an association between current smoking and RTW at 6 months postinjury. These findings suggest that smoking does not have a robust and direct association with RTW following a fracture.

Two systematic reviews have found strong evidence for physical work as a prognostic factor for RTW ^(3), (15)^, consistent with our results. The negative association between physical labor and RTW is easily understandable as a high level of activity is required when resuming physical labor. However, this factor cannot be changed by the healthcare provider as it is an occupational characteristic.

Social factors, such as nonregular employment and workers’ compensation insurance, were associated with RTW at 6 months postinjury. Receiving compensation is reportedly related to poorer outcomes after an injury, possibly due to a reduced financial imperative to recover ^(16), (17), (18)^. Furthermore, there is moderate evidence for compensation as a prognostic factor for the period of work disability ^(6)^, indicating that regular employment may encourage early RTW, whereas workers’ compensation insurance may negatively influence early RTW. Regular employment does not limit the duration of employment, contrary to nonregular employment. In general, nonregular employees are treated less favorably than regular ones in terms of insurance and salary in Japan ^(19), (20)^. As the employment contracts of nonregular employees do not guarantee a sufficient number of paid leaves when healing from a fracture, they may be laid off or resign instead. Thus, it is hoped that the government and employers will improve the treatment of nonregular employees. Moreover, according to the White Papers & Reports on Annual Health, Labor and Welfare Report 2020 issued by the Japanese government, nonregular employees have a lower income than regular ones ^(19)^, suggesting that they may not be the primary breadwinner and that they may have left the workforce as a result of injury. Nevertheless, further studies are warranted to elucidate this issue. Workers’ compensation pays not only for the patient’s medical treatment but also a portion of their salary during a patient’s leave of absence because of an injury. Social guarantees may provide the worker with adequate paid leaves, but they do not always encourage early RTW, and generous compensation may allow an excessive period of absence. Contrarily, workers’ compensation was not associated with RTW within two after injury. Paid leave is important for workers with fractures; however, a prolonged leave may be undesirable from the viewpoint of social participation. The influence of a patient’s physical condition may reasonably have a greater impact on RTW within 2 years compared with paid leaves, which are provided by worker’s accident compensation insurance.

In this study, fractures of the lower extremities were not associated with RTW at both 6 months and within 2 years postinjury. Contrarily, Sluys et al. found that minor injuries of the lower extremities have a greater impact on RTW than those of the upper extremities ^(7)^. However, minor injuries of the extremities are defined as a Triage-Revised Trauma Score of 12 (full points) ^(21)^ and an Injury Severity Score (ISS) of 2-8 ^(22), (23)^. Therefore, femoral shaft fractures with an ISS score of nine were not included in that report but were included in the present study. The difference in the RTW rate between ours and Sluys et al.’s study could be attributed to the severity of the fractures; however, further research is warranted.

Open fractures were associated with RTW at both 6 months and within 2 years after injury; this is consistent with the results of Busse et al. indicating that open fractures were associated with RTW at 1 year after a tibial fracture ^(12)^. Nonunion is more likely to occur in open fractures ^(13), (24)^, which may result in a longer treatment period. In addition, patients with nonunion fractures reportedly have a lower RTW rate than patients with union fractures ^(11)^. This may explain the negative association between open fractures and RTW in this study. Moreover, open fractures are associated with soft tissue damage, making recovery from functional impairment more difficult than that for closed fractures. Thus, increasing the rate of open fracture union and decreasing functional disability may be important for RTW.

The occurrence of FRIs combined with nonunion open fractures results in a longer healing period, which will delay RTW. When compared with a non-FRI cohort, Ilianes et al. reported that FRIs were significantly associated with prolonged absenteeism in 175 patients with FRI matched by age, sex, and fracture location (humeral, femoral, or tibial shaft) ^(25)^. Contrarily, this study demonstrated that FRIs were associated with RTW at 6 months after injury but not with that within 2 years. The possible reasons include missed diagnosis due to lower followup rates over time and the possibility that the patient developed an FRI that eventually healed during the disease course. However, this remains an issue for future studies to resolve. Nevertheless, it is important for healthcare providers to treat severe fractures while minimizing the risk of infection for early RTW.

Moderate or severe pain at 6 months postinjury was associated with RTW within 2 years; however, this pain may become chronic and prevent RTW. Surgery and trauma are frequently cited as events that trigger chronic pain. A survey of 5,130 outpatients at 10 pain clinics in the UK showed that 41% of patients attributed their chronic pain to a traumatic event or surgery, 60% reported chronic pain for more than 2 years, and 75% rated their pain as moderate or severe ^(26)^. Early fracture-related surgery has a high incidence of chronic posttraumatic/postsurgical pain (43%) ^(27)^, whereas early pain control after traumatic injury can result in a reduced risk for developing chronic pain ^(28)^. Thus, it is important to address pain at the early stages of injury as well as provide treatment to prevent infection, nonunion, or joint contracture, all of which can cause chronic pain.

Pain at 6 months after a fracture was associated with both early RTW at 6 months and within 2 years postinjury. Pain is a factor that could potentially be controlled in healthcare settings. Zomkowski et al. found that pain is a major factor preventing employees from RTW after breast cancer surgery ^(29)^ and that pain management may be similarly important for RTW following fracture surgery. Therefore, to support the RTW of patients at 6 months and within 2 years of a fracture, healthcare providers should manage and control pain in these patients.

This is the first report in Japan on RTW following musculoskeletal trauma. As such, the results serve as a landmark in the assessment of the current situation in Japan and can be used to evaluate future improvements in healthcare administration and medical care. However, this study has limitations. First, 249 of the 923 patients could not be followed up regarding RTW. This resulted in a followup rate of 73.0% at 6 months postinjury and 69.3% at 2 years, which are lower than the rate used (≥80%) when considering whether a study has a high level of evidence ^(30)^. Second, the types of occupations should be investigated and classified more accurately than simply classifying them as either physical or nonphysical work, but it was impossible due to the small number of cases in this study; thus, further research is warranted. Third, the results are based on data from only nine centers, which may not fully represent the national situation.

In conclusion, factors associated with RTW at 6 months and within 2 years after injury among workers with fractures of the long bones of the extremities were identified. Support for patients with factors that prevent RTW is desirable, and management of chronic pain may influence RTW. Treating workers with fractures and allowing them to resume work are important from the viewpoint of maintaining productivity in society. Therefore, healthcare providers, governments, and employers should assist patients in their RTW.

## Article Information

### Conflicts of Interest

None

### Sources of Funding

The study was supported by the Ministry of Health, Labor and Welfare (14080101-01) and Japan Organization of Occupational Health and Safety.

### Acknowledgement

We thank all the staff members and study participants. We would like to thank Ms. Noriko Sugano for managing the funds for this study and handling administrative communication and Mr. Yuichi Takahashi from FIRST Ltd. for their assistance.

### Author Contributions

K. Ishii. and Y. Mikami conceived the research; K. Inokuchi, T. Maehara, A. Higashikawa, Y. Sasashige, H. Konishi, F. Nakajima, Y. Tomita, S. Nobuta, and Y. Mikami collected the data; K. Ishii and H. Oka analyzed the data; K. Ishii drafted the manuscript.

### Approval by Institutional Review Board (IRB)

Code Number 5: Japan Organization of Occupational Health and Safety, Code Number 19-289: Teikyo University
